# Dysplastic L5-S1 Spondyloptosis in a 3-Year-Old Child: A Case Report and Review of the Literature

**DOI:** 10.1155/2017/1892502

**Published:** 2017-03-05

**Authors:** Vikas Tandon, Rahul Kaul, Harvinder Singh Chhabra, Ankur Nanda

**Affiliations:** Spine Department, Indian Spinal Injuries Centre, Vasant Kunj, New Delhi, India

## Abstract

A three-year-old girl presented with primary complaint of severe low back pain with radiation to both lower limbs below the knees since 2 months following history of fall and marked restriction of her daily routine activities. After clinicoradiological evaluation she was diagnosed of having dysplastic L5-S1 spondyloptosis. A staged procedure was planned after thorough discussion with her parents. During initial stage she underwent posterior decompression along L5-S1 segment including exposure of bilateral L5 and S1 nerve roots followed by instrumented reduction (L3-S2 5.5 mm pedicle screws) utilizing a rotational-translational technique. No interbody fusion was done at L5-S1 level and inner nuts of bilateral L3, L4, and S2 screws were intentionally kept loose. Subsequently after about symptom-free three-year follow up, she presented with recurrence of symptoms and underwent revision surgery as per initial plan discussed with her parents. Removals of posterior implants were done followed by stabilization with larger diameter pedicle screws (6.5 mm) at L5 and S1 level. During the same stage through anterior transperitoneal approach L5-S1 interbody fusion was done. At one-year follow-up after second-stage definitive surgery, patient remains symptom-free and fully active without any radiological evidence of reduction loss or implant failure.

## 1. Introduction

Spondyloptosis is an exceeding rare condition and more so ever publications concerning spondylolisthesis in preschool children are sparse in the existing literature, mostly in form of case reports [1]. The management of high grade lumbosacral spondylolisthesis and spondyloptosis still remains a surgical challenge involving considerable controversies [2]. We report a case of dysplastic L5-S1 spondyloptosis in a 3-year-old girl and its surgical management. To the author's best knowledge, the present case is the youngest to have undergone surgical management for L5-S1 spondyloptosis in the existing literature.

## 2. Case Report

**Figure 1:**
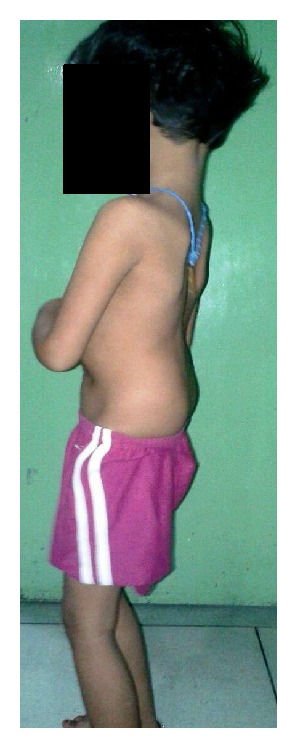
Crouched standing posture.

**Figure 2:**
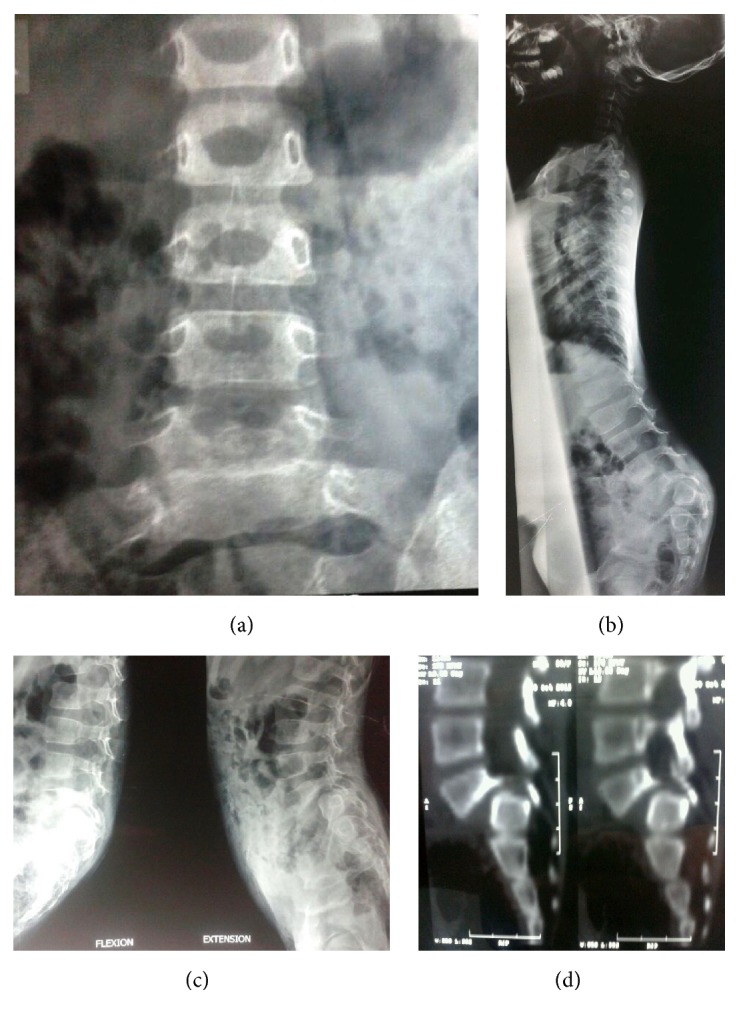
(a) Preoperative A-P radiograph lumbosacral spine. (b) Whole spine standing lateral view radiograph. (c) Flexion-extension lateral view of lumbosacral spine. (d) Sagittal reconstructed CT scan.

**Figure 3:**
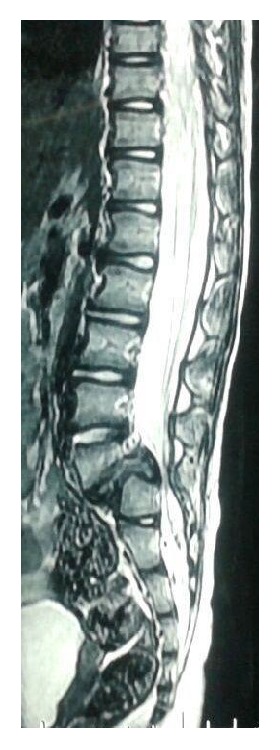
Sagittal T2 weighted MRI image.

A 3-year-old girl presented with primary complaints of severe back pain with radiation to both lower limbs below the knees 2 months following history of fall while playing as conveyed by her parents. Due to present problem she was able to walk only few steps and stand for about 5–10 minutes only. Her obstetric, developmental, and family history was normal. Inspection of her posture on standing revealed severe sagittal plane deformity including lumbosacral kyphosis and lumbar hyperlordosis with flexion at hips and knees (crouched position) (Figure 1). She had an abnormal gait pattern with a shortened stride length. Marked restriction of straight leg raise test was noted in both lower limbs because of severe hamstring spasm. Neurological examination revealed no abnormal objective neurological findings. Both lumbar flexion and extension movements were restricted with a normal range of motion of both hips and knees. Plain radiography and computed tomography (CT scan) showed L5 spina bifida occulta, bilateral L5 pars defect, dysplastic L5-S1 facets, trapezoidal L5 vertebral body, retroverted sacrum with rounding of proximal endplate, and complete anterior descent of the L5 vertebrae to the sacrum (Figures 2(a)–2(d)). Magnetic resonance imaging (MRI) of lumbosacral region revealed severe dural compression at L5-S1 level and L5-S1 intervertebral disc degeneration (Figure 3).

**Figure 4:**
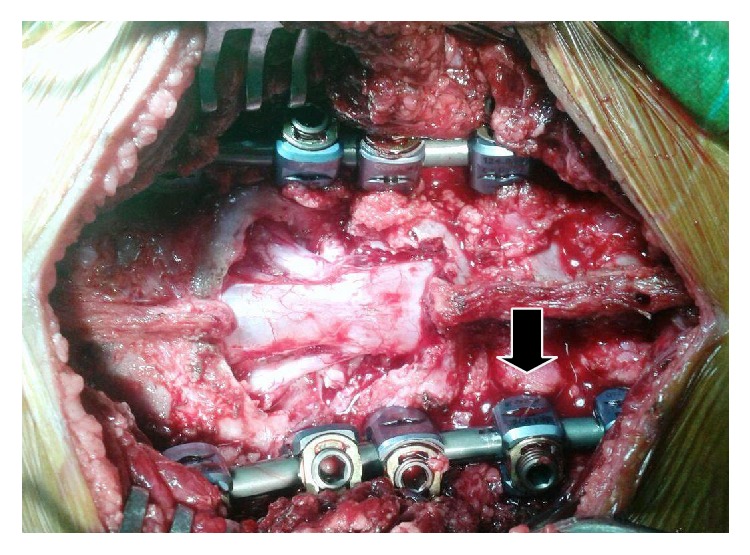
Intraoperative image (1st-stage surgery) showing loose inner nut of L4 pedicle screw (broad arrow) before final wound closure.

**Figure 5:**
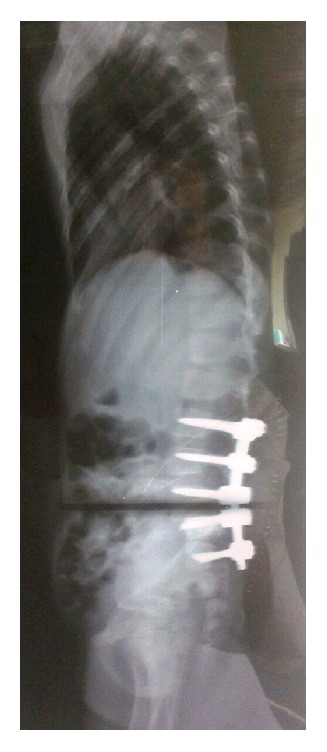
Postoperative standing lateral spine radiograph.

**Figure 6:**
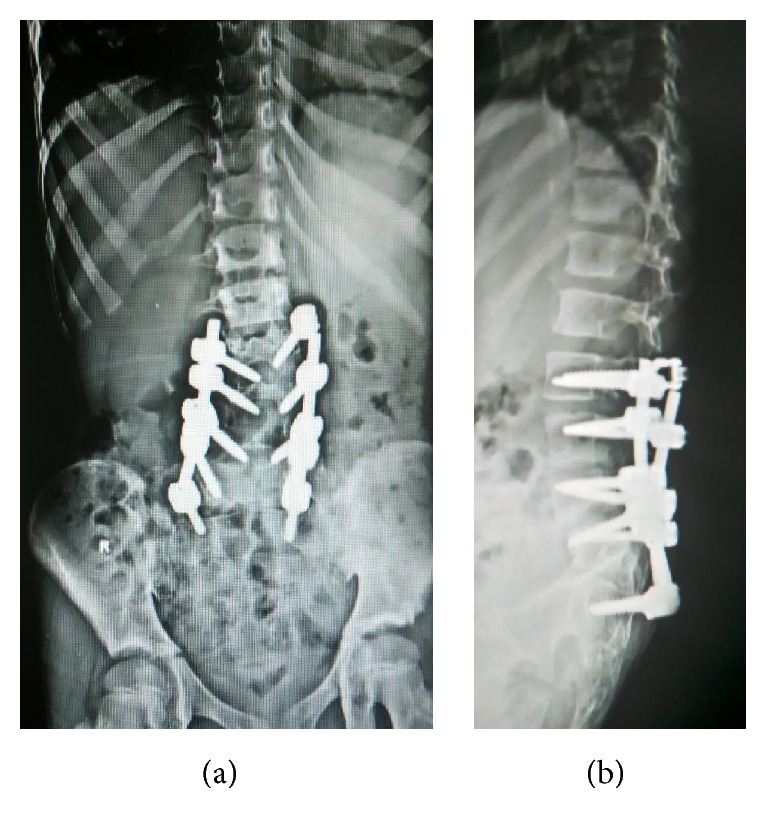
A-P and lateral radiograph of lumbosacral spine after 3-year follow-up.

**Figure 7:**
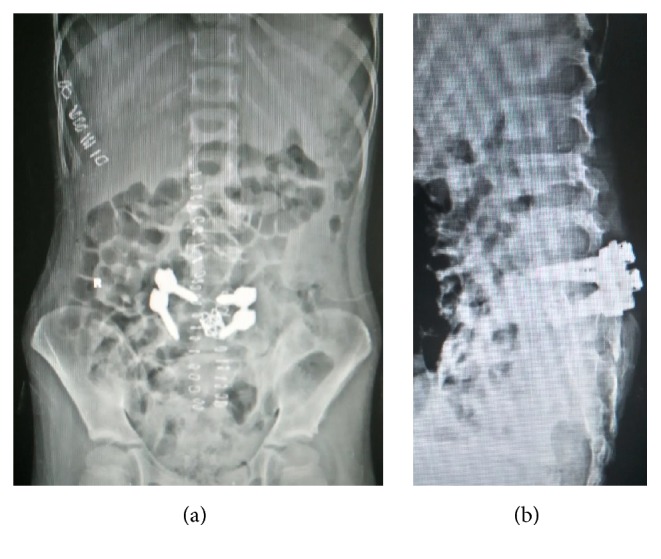
A-P and lateral radiograph of lumbosacral spine following 2nd-stage surgery.

In view of severe disability resulting from dysplastic L5-S1 spondyloptosis to the patient, surgical treatment was advocated to her parents after thorough discussion. Through a posterior approach exposure was done from L3-S2 level. On the basis of preoperative axial CT scan for the assessment of lumbosacral spine pedicle size, bilateral 5.5 mm diameter polyaxial pedicle screw insertion at L3, L4, S1, and S2 levels was done. Wide decompression was performed by removal of complete L5 lamina and superior portion of S1 lamina. Bilateral L5 and S1 roots wide foraminotomy was done, especially concerning far lateral exposure of L5 roots, and was followed by bilateral L5-S1 discectomy. Subsequently hip joints were placed in maximum extension position, which allowed a partial reduction of the pelvic retroversion and slip angle followed by a contoured rod attached on one side. A 5.5 mm diameter polyaxial reduction screw was inserted into contralateral L5 vertebrae pedicle and a contoured rod was inserted along this side. Tightening of L5 reduction screw nut was done slowly and gently resulting in pulling of its body to the rod between L4 and sacrum leading to anterior slippage correction. While performing this reduction maneuver, L5 and S1 roots were continuously observed for any undue tension or compression. Rod inserted on this side was secured by tightening of inner screw of L5 and S1 screws, and those of L3, L4, and S2 screws were kept loose (Figure 4). This was followed by contralateral L5 pedicle 5.5 mm polyaxial reduction screw insertion after rod removal, and finally a contoured rod was secured this side in a similar manner as done on the opposite side. Length of 5.5 mm diameter screws inserted from L3 to S1 level was in the range of 30–35 mm, whereas both S2 pedicle screws' tip was cut to a length of about 25 mm. All screws were inserted in a convergent manner in order to reduce the risk of screw pull out during reduction maneuver. Neuromonitoring including stimulus evoked EMG monitoring of L5 and S1 roots was used during the procedure followed by wake-up test at the end, and no neurological deficit was observed especially concerning bilateral L5 and S1 nerve roots. Postoperatively both hip and knee were maintained in slight flexion and were gradually extended subsequently. After about one week patient was made to stand and mobilized without any lumbar support. Objective radiological measurements performed after surgery, which included percent displacement, slip angle, sacral inclination, total lumbar lordosis (L1–L5), and global sagittal balance (plumb line from centre of C7 in relation to posterosuperior edge of sacrum), showed marked improvement on comparison to presurgery measurements (Figure 5). At the time of discharge, the subsequent need of the anterior L5-S1 interbody fusion in view of implant loosening and reduction loss during future course was explained to the patient's parents. Till the last follow-up of about 36 months done at regular interval of 3–6 months including radiological evaluation, patient maintained her activity level much improved compared to her presurgery level without any significant pain or neural deficit. On her subsequent visit following the above-mentioned time interval, patient complained of lower back pain which increased progressively with her routine activities. Radiological evaluation revealed disengagement of screw rod complex at proximal end of construct along left side and loosening with screw back out of S2 pedicle screws along lower end of the screw assembly (Figures 6(a) and 6(b)). Following this patient was taken for subsequent operation, as was discussed earlier with her parents following previous surgery. Following initial exposure through posterior approach all pedicle screws were removed initially on left side, and 6.5 mm diameter polyaxial pedicle screws were inserted at L5 and S1 level followed by attaching them to a contoured rod. Similar procedure was done on contralateral side. Subsequently under the same anesthesia through anterior transperitoneal lumbar approach L5-S1 level was exposed for performing interbody fusion. After preparing the end plates appropriate size titanium mesh cage was inserted, incorporated with bone graft obtained after harvesting autogenous rib (Figures 7(a) and 7(b)). Following conclusion of the revision surgery, wake-up test was performed and patient maintained her intact preoperative neurological status especially concerning bilateral L5 nerve root status.

**Figure 8:**
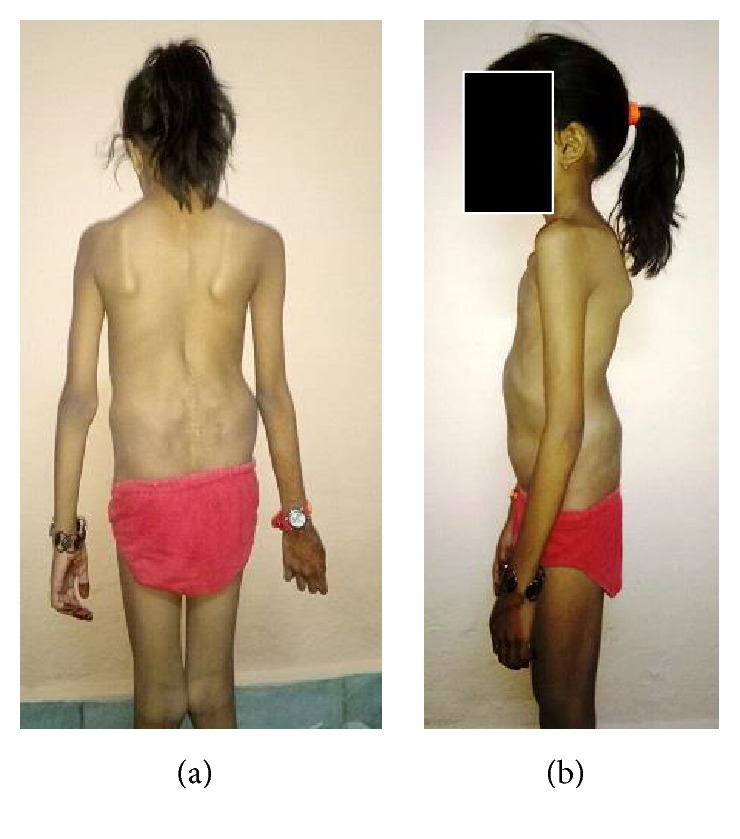
A-P and lateral clinical photograph one year postoperatively.

During the most recent follow-up of about 12 months following the subsequent surgery, patient is pain-free and fully active and on clinical evaluation her spinal alignment was found to be appropriate (Figures 8(a) and 8(b)). Radiological evaluation revealed no reduction loss and nonunion or loosening of graft and breakage of hardware.

## 3. Discussion

High grade spondylolisthesis and spondyloptosis usually occur in dysplastic type involving lumbosacral region, although it has been stated in the existing literature that progression of slip beyond 25 percent is not possible without concomitant pars defect [3]. According to Marchetti and Bartolozzi classification, high dysplastic developmental spondylolisthesis (HDDS) is characterized by three main pathological conditions: anterior slippage of L5 against S1, segmental kyphosis of L5-S1 segment, and sacral retroversion [4]. The typical posture in HDDS patients occurs due to local deformity resulting from pelvis retroversion causing hyperextension of hip joints, and lumbosacral kyphosis leading to compensatory hyperlordosis of adjacent lumbar segment, as was seen in our case [5]. The precise factors determining spondylolisthesis progression and its rate are still unknown in the existing literature [6]. Yue et al. suggested that severe growth plate damage in the immature proximal sacrum plays a central role for spondyloptosis to occur, whereas other features have only facilitative role [3].

Generally surgical management is the accepted treatment for the high grade developmental spondylolisthesis [7]. However there still exists controversy regarding the need for reduction, extent of reduction, and surgical technique required for management of high grade developmental spondylolisthesis [8]. According to recent Spinal Deformity Study Group (SDSG) classification for lumbosacral spondylolisthesis mandatory reduction in high grade deformities with an unbalanced sacropelvic and spinopelvic balance is required, as was in our case [9].

Posterior in situ fusion without reduction usually performed between L4 and S1 vertebrae has low rate of neurological complication combined with minimal operative disruption [8]. Although good clinical results have been reported by some, this procedure has serious disadvantages: High pseudarthrosis rate, postoperative progression by plastic bone remodeling despite consolidated fusion mass, and even neurological compromise as a late sequel [7, 10–12]. Due to these reasons we preferred not to perform this procedure.

Although there exists controversy regarding reduction for the treatment of spondyloptosis, it helps to restore segmental lordosis and correct sacral position which normalize the overall sagittal profile [2]. In the existing literature various closed and surgical reduction methods have been described including casting, traction, and reduction with specialized instrumentation and fusion by anterior, posterior, or combined approaches [8]. The reduction maneuver performed in our case was similar to the technique described by Lamartina et al. [5]. Firstly removal of constraints resulting in deformity stiffness at lumbosacral junction was achieved by performing a wide posterior decompression including release of bilateral L5 and S1 nerve roots far lateral from their foramen and bilateral excision of L5-S1 disc. Subsequent hyperextension of hip joints provided anterior rotation forces to the pelvis, resulting in a partial reduction of pelvic retroversion and slip angle. Finally gradual progressive traction forces applied through polyaxial reduction pedicle screws into L5 vertebrae were able to overcome residual stiffness, allowing translation of L5 vertebrae posteriorly. The path of L5 vertebrae reduction in a dome shaped sacrum is primarily rotational, resulting in a more significant L5-S1 kyphosis correction than in a flat sacrum; this may have further helped in our case [13].

It has been suggested that reduction of slip in high grade spondylolisthesis produces a relative anterior column deficiency due to inability of abnormal lumbosacral disc to share load in the reduced position, highlighting the need of anterior column grafting [7]. Considering the possibility of decreased longitudinal growth of the spinal column at L5-S1 level secondary to destruction of their ring apophysis, chances of developing spinal canal stenosis due to suppression of neurocentral cartilage (NCC) growth through tethering effect of anterior spinal fusion in child before six years of age, development of secondary deformities, and restricted range of motion, we did not perform interbody fusion during the initial surgery in our case [14, 15]. It was performed during the subsequent surgery following loosening of the implant, when child was already more than six years of age.

Our decision to use pedicle instrumentation in a 3-year-old child after preoperative CT scan evaluation was based on the studies which have established their safe use in very young children without vertebral growth retardation [16, 17]. Ruf et al. proposed that, in comparison to laminar hooks, pedicle screws allowed application of higher corrective forces in very young patients with soft lamina, another reason which further reinforced our decision [8]. Although pedicle screw instrumentation was extended from L3 to S2 level in order to withstand significant biomechanical stresses resulting from reduction of dysplastic L5-S1 spondyloptosis slip, inner screws of L3, L4, and S2 screws were kept loose. This was done for permitting the screws to slide along the rods as these vertebral segments would achieve their longitudinal growth similar to the Shilla growth guidance technique for early onset spinal deformities, without significantly compromising the stability of the implant construct [18].

The most serious complication of spondylolisthesis reduction is the iatrogenic neurological injury and it has shown to correlate with degree of reduction achieved [19]. Intraoperative measures which may have prevented this complication from occurring included wide exposure of L5 and S1 roots with special concern for far lateral exposure of L5 roots, continuous visualization of L5 and S1 roots during the reduction maneuver for any compression or undue tension, avoiding excessive distraction and gradual reduction to allow viscoelastic stress relaxation of tissues, and use of neuromonitoring including stimulus evoked EMG monitoring followed by wake-up test after the completion of reduction maneuver [8, 20]. During the immediate postoperative period, patient was gradually mobilized in order to prevent the occurrence of delayed neurological deficit [10]. Fabris et al. proposed regarding plasticity of the neural structures in young patients which prevents them from serious damage during reduction, another factor which may have contributed to our case also [20].

On literature review concerning surgical management of L5-S1 spondyloptosis in preschool children (3–5 years), only one case report involving single case by Wild et al. was found [21] (Table 1). Although the problem was detected by the authors when child was 18 months old, he underwent surgical management at 5 years of age. During initial surgery three-stage procedures (back-front-back) were done: L5 lamina resection and wide L5 nerve root decompression, anterior subtotal resection of inferior L5 body with interbody morcellized vertebral body graft between L5 and S1, and finally posterior instrumentation (L2-S1, sacral Cotrel-agraffe device) with reduction of L5-S1 spondyloptosis. Subsequently after about 8 months, posterior instrumentation was removed; decortication of superior articular facets and both transverse processes of L5 vertebrae was done. Autogenous bone graft from iliac crest was taken and inserted to augment previous fusion. To the author's best knowledge, the present case is the youngest to have undergone surgical management for L5-S1 spondyloptosis in the existing literature.

## 4. Conclusion

Although this is only a single case being the first to our knowledge, it highlights the strategy to tackle one of the most challenging pathologies faced by the spinal surgeons worldwide involving rare dysplastic L5-S1 spondyloptosis in a very young child. We recommend a staged procedure: first stage involving a rotation-translation technique for deformity correction to improve overall sagittal profile utilizing pedicle screw instrumentation which permits subsequent longitudinal growth of the involved spinal segments and second stage providing interbody support resulting in final fusion, thus reducing the growth potential loss and possibility of secondary deformity in the involved segments. However long term follow-up is necessary to determine effects of this surgical approach.

## Figures and Tables

**Table 1:** Literature review regarding management of L5-S1 spondyloptosis in preschool children (3–5 years).

Authors	Number of cases	Age (year)/sex	Management
Wild et al. [21]	1	5 yr/male	During initial surgery three-stage procedure (back-front-back). L5 lamina resection and wide L5 nerve root decompression, anterior subtotal resection of inferior L5 body with interbody morcellized vertebral body graft between L5 and S1, and finally posterior instrumentation (L2-S1) with reduction of L5-S1 spondyloptosis. Subsequently after about 8 months, posterior instrumentation was removed; decortication of superior articular facets and both transverse processes of L5 vertebrae was done. Autogenous bone graft from iliac crest was taken and inserted to augment previous fusion.

Our case	1	3 yr/female	Wide posterior decompression followed by 5.5 mm pedicle instrumentation from L3-S2 vertebrae including bilateral 5.5 mm polyaxial reduction screw for L5 vertebrae and spondyloptosis reduction via rotation translation technique. Subsequently inner nuts of bilateral L3, L4, and S2 pedicle screws were kept loose and no interbody graft was used. Finally following implant loosening after about >3-year follow-up, posterior instrumentation was revised with 6.5 mm polyaxial pedicle screws at L5 and S1 levels. Same stage anterior interbody L5-S1 fusion by transperitoneal approach.
